# Prenatal Diagnosis and Molecular Cytogenetic Characterization of Copy Number Variations on 4p15.2p16.3, Xp22.31, and 12p11.1q11 in a Fetus with Ultrasound Anomalies: A Case Report and Literature Review

**DOI:** 10.1155/2020/1761738

**Published:** 2020-05-27

**Authors:** Han Zhang, Qi Xi, Xiangyin Liu, Fagui Yue, Hongguo Zhang, Meiling Sun, Ruizhi Liu

**Affiliations:** ^1^Center for Reproductive Medicine, Center for Prenatal Diagnosis, First Hospital, Jilin University, Changchun 130021, China; ^2^Jilin Engineering Research Center for Reproductive Medicine and Genetics, Jilin University, Changchun 130021, China

## Abstract

Chromosomal rearrangements, such as duplications/deletions, can lead to a variety of genetic disorders. Herein, we reported a prenatal case with right aortic arch and aberrant left subclavian artery, consisting of a complex chromosomal copy number variations. Routine cytogenetic analysis described the chromosomal karyotype as 46,XY, add (2)(q37) for the fetus. However, the chromosomal microarray analysis (CMA) identified a 22.4 Mb duplication in chromosome 4p16.3p15.2, a 3.96 Mb microduplication in 12p11.1q11, and a 1.68 Mb microdeletion in Xp22.31. Fluorescence in situ hybridization (FISH) using a chromosome 4 painting probe was found to hybridize to the terminal of chromosome 2q on the fetus, thus confirming that the extra genetic materials of chromosome 2 was actually trisomy 4p detected through CMA. Meanwhile, the parental karyotypes were normal, which proved that the add (2) was *de novo* for fetus. The duplication of Wolf-Hirschhorn syndrome critical region (WHSCR) and X-linked recessive ichthyosis associated with Xp22.31 deletion separately were considered potentially pathogenic causes although other abnormalities involving these syndromes were not observed. For prenatal cases, the combined utilization of ultrasonography, traditional cytogenetic, and molecular diagnosis technology will enhance better diagnostic benefits, offer more detailed genetic counselling, and assess the prognosis of the fetuses.

## 1. Introduction

Trisomy 4p syndrome, partial or almost duplication of the short arm in chromosome 4, is a rare chromosomal anomaly in clinic [1]. Since it was initially described as a distinct clinical entity in the 1970s, more than 80 cases of trisomy 4p had been reported [1, 2]. Most trisomy 4p cases involve at least the distal half of 4p (i.e., 4p14-4pter) or more [3]. Trisomy 4p has been associated with various clinical symptoms, such as growth retardation, psychomotor delay, mental retardation, and dysmorphic features such as facial dysmorphism, microcephaly, skeletal abnormalities, and heart defects [2, 4]. The formation mechanism of trisomy 4p usually results from an unbalanced segregation of a parental balanced translocation or a *de novo* duplication [4]. Currently, it is difficult to establish clear genotype-phenotype correlations for trisomy 4p cases that usually exhibit different sizes of duplicated region accompanied by another chromosomal deletion [2].

Xp22.3 microdeletion is associated with several genetic syndromes in males, which includes short stature, X-linked recessive chondrodysplasia punctata, X-linked mental retardation, X-linked ichthyosis, and Kallmann syndrome [5, 6].

Trisomy 12 is a rare chromosomal disorder resulting in spontaneous abortion easily. Partial duplications are associated with diverse clinic phenotypes, ranging from normal phenotypes to severe physical defects in different organ systems [7].

Here, we report a prenatal case with abnormal sonography findings, consisting of a *de novo* 4p15.2p16.3 duplication translocated to the terminal of the long arm in chromosome 2, a Xp22.31 microdeletion and a 12p11.1q11 duplication. Meanwhile, we also compare the clinical features of the cases involving similar 4p duplication as described in the literature and databases.

## 2. Materials and Methods

### 2.1. Patient

A 23-year-old, gravida 1, para 0, woman underwent amniocentesis for cytogenetic and chromosomal microarray analysis (CMA) at 25 weeks of gestation due to the right aortic arch and aberrant left subclavian artery in prenatal sonography findings. The pregnant woman and her husband were nonconsanguineous and healthy. They both showed no family history of diabetes mellitus or congenital malformations. The wife denied any exposure to alcohol, teratogenic agents, irradiation, or infectious diseases during this pregnancy. Our study protocol was approved by the Ethics Committee of the First Hospital of Jilin University (No. 2019-261), and written informed consent was obtained from the couple.

### 2.2. Cytogenetic Analysis

Cytogenetic studies were carried out on G-band metaphases collected from cultured amniotic fluid cells and peripheral blood cells of the couple. Chromosome preparations were obtained according to G-banding technique with a resolution between 300 and 400 bands. We analyzed twenty metaphases for the fetus and the couple. And the chromosomal karyotypes were described according to the ISCN 2016 nomenclature [8].

### 2.3. Chromosomal Microarray Analysis (CMA)

Following written consent from the pregnant woman, 10 mL of uncultured amniotic fluid cells was collected through amniocentesis. Genomic DNA was isolated using Qiagen micro kit with the manufacturer's protocol. Then, the procedures are conducted through CytoScan 750K array (Affymetrix, Santa Clara, CA, USA), in accordance with the manufacturer's protocol and our previous study [9]. The procedure included genomic DNA extraction, digestion and ligation, PCR amplification, PCR product purification, quantification and fragmentation, labeling, array hybridization, washing, and scanning. Thresholds for genome-wide screening were set at ≥200 kb for gains and ≥100 kb for losses. The detected copy number variations were comprehensively estimated by comparing them with published literature and the public databases: (1) Database of Genomic Variants (DGV) (http://dgv.tcag.ca/dgv/app/home), (2) DECIPHER (http://decipher.sanger.ac.uk/), (3) ISCA https://www.iscaconsortium.org/), (4) ECARUCA (http://www.ecaruca.net), and (5) OMIM (http://www.ncbi.nlm.nih.gov/omim).

### 2.4. Fluorescence In Situ Hybridization (FISH)

Based upon the results of karyotype analysis and CMA results, fluorescence in situ hybridization (FISH) using a whole chromosome painting probe specific for chromosome 4 (Cytocell Technologies, Cambridge, UK) was performed on metaphase slides for the fetus to confirm the existence of trisomy 4p, according to the manufacturer's protocol.

## 3. Results

**Figure 1:**
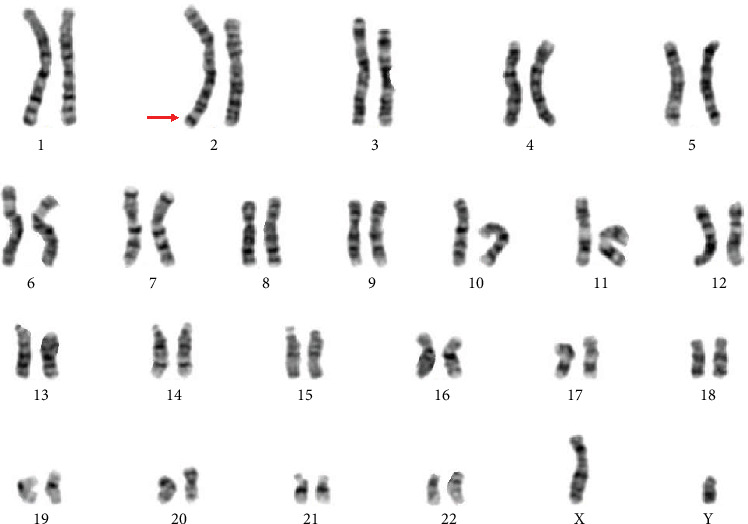
The karyotype of the fetus identified by GTG banding technique: 46,XY, add (2)(q37). Arrow indicated the sSMC.

**Figure 2:**
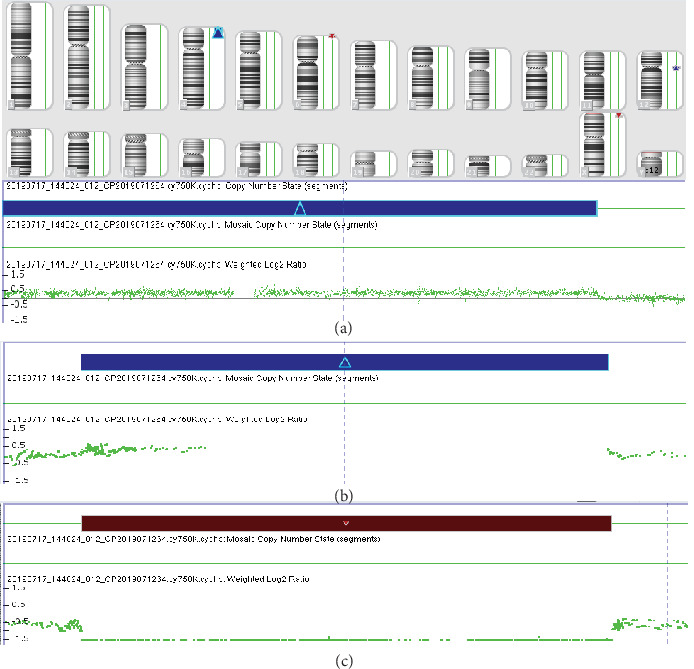
The CMA results depicted a 22.4 Mb gain of the chromosome 4p16.3p15.2 (a), a 3.96 Mb gain of the chromosome 12p11.1q11 (b), and a 1.68 Mb loss of the chromosome Xp22.31 (c) for the fetus.

**Figure 3:**
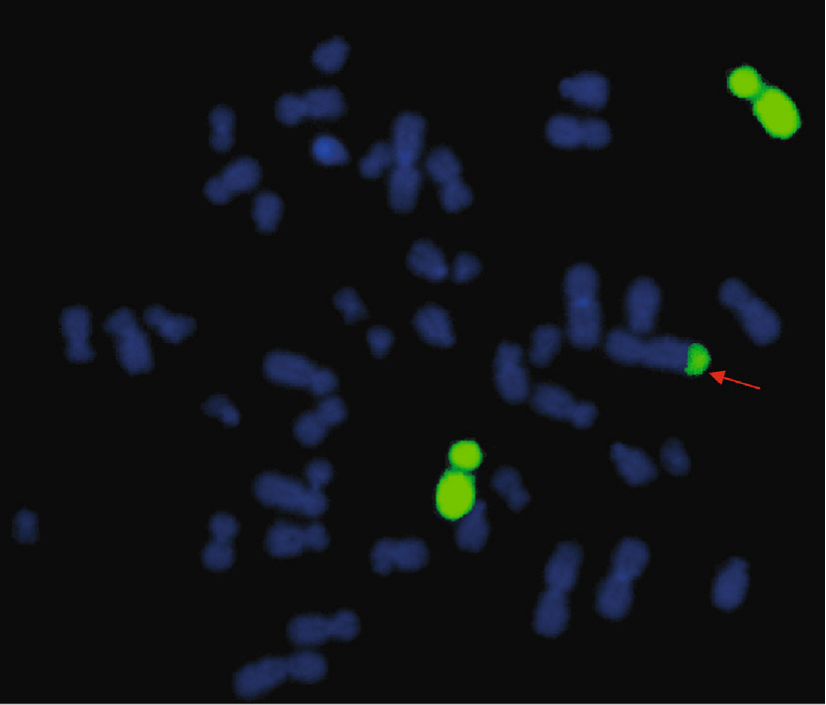
Metaphase-FISH results of whole chromosome 4 (green) painting probe for the fetus. Arrow indicates partially duplicated chromosome 4 attached on the long arm of chromosome 2.

The routine cytogenetic analysis described the chromosomal karyotype as 46,XY, add (2)(q37) (Figure 1). However, the CMA detection identified a complex chromosomal copy number variations (CNVs): arr [GRCh37] 4p16.3p15.2(68345-22489538)×3; arr [GRCh37] 12p11.1q11 (33909111-37869107)×3; and arr [GRCh37] Xp22.31 (6455151-8135568)×0 (Figure 2). It was inferred that the extra genetic materials of chromosome 2q might be the partial 4p duplication. Based upon the speculation, we informed the couple that peripheral chromosome studies will be performed to confirm that add (2) was parentally inherited or *de novo*. Meanwhile, the whole chromosome painting probe specific for chromosome 4 was applied to confirm whether the segment of trisomy 4p was located on chromosome 2. The G-banding analysis demonstrated that the parental karyotypes were normal, which illustrated that the add (2) in the fetus was *de novo*. FISH using chromosome 4 painting probe was found to hybridize to the terminal of chromosome 2q on the fetus, thus confirming that the existence of add (2) was actually the 4p duplication detected through CMA (Figure 3). The couple finally chose to terminate the pregnancy according to genetic counselling based upon abnormal cytogenetic and molecular genetic analysis.

## 4. Discussion

In our study, we report a prenatal case with right aortic arch and aberrant left subclavian artery in ultrasonography. The fetus carried a 4p16.3p15.2 duplication attached to the terminal of chromosome 2q, a Xp22.31 microdeletion, and a 12p11.1q11 microduplication, which were identified by the combined application of G-banding technique, CMA, and FISH analysis. To the best of our knowledge, these chromosomal complex rearrangements have not been reported previously.

Trisomy 4p, as a rare chromosomal disorder, is a distinct syndrome due to various degrees of duplicated regions in the short arm of chromosome 4 [10]. Trisomy 4p cases usually presented growth retardation and psychomotor retardation with or without seizures, as well as various major and minor anomalies, including microcephaly, prominent glabella, bulbous nose, retrognathia, pointed chin, short neck, enlarged ears, rocker-bottom feet, arachnodactyly, and camptodactyly [2, 11].

To better interpret the phenotype-karyotype correlations, we summarized the clinical manifestations of cases with similar/overlapping 4p15.2p16.3 duplication according to literature review and databases, as shown in Table 1 [12–17]. The age of the patients ranged from 2 months to 27 years. Among these duplications, 4/10 were parentally inherited, 3/10 patients were *de novo*, and 3/10 patients were not available. The high frequencies of different clinical manifestations were as follows: ear anomaly (7/10), growth retardation (6/10), limb anomaly (6/10), intellectual disability (5/10), psychomotor retardation (5/10), short neck (4/10), widely spaced nipples (3/10), and microcephaly (3/10). It was worth noting that varying degrees of abnormal craniofacial features could be observed in all cases. To our knowledge, right aortic arch and aberrant left subclavian artery were not reported in prenatal cases with 4p15.2p16.3 duplication before. Unbalanced segregation of a parental balanced translocation or inversion involving chromosome 4 would usually lead to partial trisomy 4p, accompanied by another chromosome or 4q monosomy [18], and the karyotypes of 6/10 cases in our review were consistent with this description. The couple accepted chromosomal karyotypic analysis to testify whether the imbalanced genomic aberrations of the fetus resulted from parentally balanced chromosomal rearrangement or not. The normal karyotypes of the couple illustrated that the add (2) in the fetus was *de novo*. Afterwards, molecular genetic analysis proved the existence of 4p duplication, which was translocated to the terminal of chromosome 2q. Generally speaking, more research should be gathered to describe a distinct phenotype-karyotype correlation on pure 4p duplication.

Chromosomal rearrangements, such as duplications/deletions, can result in a series of genetic diseases [19]. Dosage-sensitive genes, due to the chromosomal duplication or deletion, could result in a wide range of clinic phenotypes, including heart disease, cancers, and neuropsychiatric disorders [20]. Based upon the DECIPHER database, the morbid genes located in the three regions and their associated diseases are summarized in Table 2. According to the literature review and databases, we delineated the potentially pathogenic genes probably exhibiting a “dose effect” and related duplication/deletion syndrome, which could predict the possible phenotypes in clinic and poor prognosis for the fetus.

In our study, the CMA detected a duplication of 4p15.2p16.3 containing the critical 4p16.3 region, the deletion of which was associated with Wolf-Hirschhorn syndrome (WHS; OMIM: 194190), first described in the 1960s [21]. Patients with this deletion usually presented growth impairment, intellectual disability, congenital malformations, distinctive craniofacial appearance, and seizures [22, 23]. Two critical regions are involved in the Wolf-Hirschhorn syndrome critical region (WHSCR): WHSCR1 (WHSC1 and WHSC2 genes included) and WHSCR2 (WHSC1 and LETM1 genes included). The gene WHSC1 (OMIM: 602952) is primarily associated with the clinic features of Wolf-Hirschhorn syndrome, including developmental retardation and abnormal facial appearance. And the gene LETM1 (OMIM: 604407) is proposed to be responsible for seizures in WHS patients [24]. However, more investigations revealed that trisomy 4p patients with WHSCR also presented development/psychomotor retardation and craniofacial and skeletal malformations [2, 18]. Patients with half 4p duplication were characterized by multiple congenital anomalies, mental retardation, broad nasal bridge, and ear anomalies [25], and 4p16 duplication has been correlated with overgrowth and mental retardation [26]. According to the ClinGen database, there is some evidence for the triplosensitivity in association with the WHSCR duplication. Although the fetus in our report only showed right aortic arch and aberrant left subclavian artery at present, it could be speculated that other abnormal symptoms might gradually appear in the late pregnancy or after birth.

In our study, the CMA detected a 1.68 Mb deletion in Xp22.31, a most genomic instable region in the short arm of chromosome X. The genes in this deleted region included STS, HDHD1, VCX, and PNPLA4. STS (OMIM: 300747), located in Xp22.31, encodes steroid sulfatase as metabolic precursors for estrogens, androgens, and cholesterol. The deletion of this causative gene is closely associated with X-linked recessive ichthyosis (XLI; OMIM: 308100), which is a typically inherited disorder characterized by scaly skin on the scalp, trunk, neck, and extremities after birth. The incident rate is about 1/1500 in males, and approximately 90% XLI patients are involved in STS gene deletion [27]. During the process, the deficiency of the enzyme steroid sulfatase (STS) is critically responsible for abnormal phenotypes [28]. According to the ClinGen database, there is sufficient pathogenic evidence for haploinsufficiency associated with STS. Based upon the DECIPHER database and published literature, intellectual disability and learning difficulties can also be observed in patients with XLI. Xp22.3 deletion in XLI males can be associated with mental retardation, which should be taken seriously [29]. HDHD1 (OMIM: 306480), often missing in XLI, encodes a pseudouridine-5′-phosphatase. It is specifically involved in dephosphorylation of a modified nucleotide present in RNA [30]. PNPLA4 (OMIM: 300102) might play a role in regulating epidermal homeostasis [31]. The VCX (OMIM: 300229) gene might not be associated with intellectual development, while potential correlation between the VCX gene and cognitive development and/or communication still requires further research [32]. Based upon the above, the male fetus was recognized as a potential XLI patient.

In addition, a duplication in 12p11.1q11 was detected. ALG10 (OMIM: 618355), encoding a membrane-associated protein, might be associated with nonsyndromic hearing impairment in mice. Currently, there is no pathogenic evidence on ALG10 duplication [33].

For visible chromosomal anomalies of unknown origin, traditional cytogenetic analysis can hardly identify the aberrations due to the low banding resolution, but the application of CMA could detect the extra or missing genetic materials quickly and assess the clinic significance through genome browsers and public databases, which offers a more precise genetic counselling for these patients [34, 35]. In our study, 4p15.216.3 duplication involving WHSCR and Xp22.31 deletion associated with XLI were detected simultaneously in the fetus, which might lead to unavoidable poor prognosis, so the couple finally chose termination of pregnancy. Considering normal karyotypes of the couple, CMA should also be carried out for the couple before they intend to conceive again. Considering the possibility of the mother being a Xp22.32 deletion carrier, preimplantation genetic testing could be considered. Equally, prenatal diagnosis is essential after the wife gets pregnant.

## 5. Conclusions

In our study, we delineate a prenatal case with ultrasound findings, consisting of a *de novo* partial 4p15.2p16.3 duplication, a Xp22.31 microdeletion, and a 12p11.1q11 microduplication. Among the three CNVs, 4p15.2p16.3 was translocated to the terminal of the long arm in chromosome 2, which was testified by FISH. Although no other abnormalities were observed, WHSCR duplication and X-linked recessive ichthyosis involved in the critical duplicated/deleted regions were considered pathogenic factors. For prenatal cases, the combined utilization of prenatal ultrasound screening and traditional cytogenetic and molecular genetic analysis will exert better diagnostic benefits and offer more significant cytogenetic information, so more precise genetic counselling could be given in clinic.

## Figures and Tables

**Table 1:** Summary of clinical manifestations in patients with similar/overlapping duplication in 4p15.2p16.3.

References	Wu [12]	Garcia-Heras [13]	Hirsch and Baldinger [14]	Bartocci et al. [15]	Kim et al. [16]		Maurin et al.[17]	DECIPHER 308723	DECIPHER 395372	DECIPHER 296428	Present case
					Case 1	Case 2					
Age/sex	6/M	4/F	27/F	22/F	2 m/F	9/F	2/F	<1/M	27/F	N.R./M	Fetus/M
Gestational age	39 w	36 w	N.R.	At term	38 w + 3 d	39 w + 5 d	At term	N.R.	N.R.	N.R.	TOP
Birth weight (g)	3,800	N.R.	N.R.	N.R.	2,600	2,600	3,290	N.R.	N.R.	N.R.	N.A.
Duplicated region	4p15.33-p16.3	4p15-4pter	4p15.32-4pter	4pter-4p15.2	4pter-4p15.31	4pter-4p15.31	4p15.1-4pter	4p15.3-14pter	4p15.2-4pter	4p15.2-4pter	4p15.2-p16.3
Karyotype	46, XY, add (20) (q13.3) Mother: 46, XX, t(4;20)(p15.2; q13.1)	46,XX,rec(4)dup4p inv(4)(p15q35) mat	46,XX,rec(4)dup p,inv(4)(p15.32q35)mat	46,XX,del(X)(q27→qter)	47,XX,+mar[44]/46, XX [6] Mother: 46,XX,t(4;14)(p15.3;q12)	47,XX,+mar Mother: 46,XX,t(4;14)(p15.3;q12)	46,XX,rec(4)dup(4p)inv(4)(p15.1q35.1)pat	N.R.	N.R.	N.R.	46,XY,add (2)(q37) *De novo*
Intellectual disability	+	+	+	N.R.	N.R.	N.R.	N.R.	N.R.	+	+	Ultrasonic indication: right aortic arch and aberrant left subclavian artery
Growth retardation	-	+	+	+	+	+	+	N.R.	N.R.	N.R.	
Psychomotor retardation	+	+	N.R.	+	N.R.	+	+	N.R.	N.R.	N.R.	
Language retardation	+	+	N.R.	N.R.	N.R.	N.R.	N.R.	N.R.	N.R.	N.R.	
Seizures	+	N.R.	N.R.	+	N.R.	N.R.	N.R.	N.R.	N.R.	N.R.	
Hypotonia	N.R.	+	N.R.	N.R.	N.R.	N.R.	+	N.R.	N.R.	N.R.	
Microcephaly	N.R.	+	+	N.R.	N.R.	N.R.	+	N.R.	N.R.	N.R.	
Face	N.R.	+	Asymmetry	Asymmetry	N.R.	Round	Abnormal	N.R.	Asymmetry	Abnormal	
Nasal bridge	Prominent	Flat	N.R.	Broad	N.R.	N.R.	N.R.	Depressed/wide	N.R.	N.R.	
High arched palate	N.R.	+	N.R.	+	N.R.	N.R.	N.R.	N.R.	N.R.	N.R.	
Epicanthus	+	N.R.	N.R.	+	N.R.	N.R.	N.R.	N.R.	N.R.	N.R.	
Ptosis	N.R.	N.R.	+	N.R.	N.R.	N.R.	N.R.	N.R.	+	N.R.	
Palpebral fissures	N.R.	N.R.	N.R.	Short	Short	N.R.	Down-slanting	N.R.	N.R.	N.R.	
Ears anomaly	Low-set	-	Prominent	Rotated	Low-set	Small	+	+	N.R.	N.R.	
Short neck	N.R.	+	N.R.	+	N.R.	+	+	N.R.	N.R.	N.R.	
Limb anomaly	N.R.	-	+	+	-	+	+	+	+	N.R.	
Widely spaced nipples	N.R.	+	N.R.	N.R.	N.R.	N.R.	+	+	N.R.	N.R.	
Others	Tall stature, macrocephaly	Bulbous nasal tip, large mouth, short philtrum, bowed upper lip, gingival hypertrophy		Hypertelorism, bushy eyebrows, low frontal hairline, micrognathia		Asymmetric eyes	Anteverted nostrils, large philtrum, thin upper lip, interventricular and auricular septal defects	Broad forehead, thin vermilion border	Scoliosis		

F: female; M: male; m: months; N.R.: not reported; TOP: termination of pregnancy.

**Table 2:** Morbid genes in the region of 4p15.2p16.3 and Xp22.31 and the associated diseases.

Gene	Location	OMIM	Description	Disease
ZNF141	4p16.3	194648	Zinc finger protein 141	Polydactyly, postaxial, type A6
PIGG	4p16.3	616918	Phosphatidylinositol glycan anchor biosynthesis class G	Mental retardation, autosomal recessive 53
PDE6B	4p16.3	180072	Phosphodiesterase 6B	Night blindness, congenital stationary, autosomal dominant 2, retinitis pigmentosa-40
CPLX1	4p16.3	605032	Complexin 1	Epileptic encephalopathy, early infantile, 63
SLC26A1	4p16.3	610130	Solute carrier family 26 member 1	Nephrolithiasis, calcium oxalate
IDUA	4p16.3	252800	Iduronidase alpha-L-	Mucopolysaccharidosis Ih, mucopolysaccharidosis Ih/s, mucopolysaccharidosis Is
RNF212	4p16.3	612041	Ring finger protein 212	Recombination rate QTL 1
CTBP1	4p16.3	602618	C-terminal binding protein 1	Hypotonia, ataxia, developmental delay, and tooth enamel defect syndrome
UVSSA	4p16.3	614632	UV stimulated scaffold protein A	UV-sensitive syndrome 3
FGFR3	4p16.3	134934	Fibroblast growth factor receptor 3	Achondroplasia, bladder cancer, somatic, colorectal cancer (CRC), hypochondroplasia, LADD syndrome, nevus, epidermal, thanatophoric dysplasia, type I, thanatophoric dysplasia, type II, testicular germ cell tumor (TGCT), Muenke syndrome, cervical cancer, somatic, CATSHL syndrome, Crouzon syndrome with acanthosis nigricans, SADDAN
NAT8L	4p16.3	610647	N-Acetyltransferase 8 like	N-Acetylaspartate deficiency
HTT	4p16.3	613004	Huntingtin	Huntington disease, lopes-Maciel-Rodan syndrome
DOK7	4p16.3	610285	Docking protein 7	Myasthenic syndrome, congenital, 10, fetal akinesia deformation sequence 3
SH3BP2	4p16.3	602104	SH3 domain binding protein 2	Cherubism
ADD1	4p16.3	102680	Adducin 1	Hypertension, essential
LRPAP1	4p16.3	104225	LDL receptor-related protein-associated protein 1	Diseases: myopia 23, autosomal recessive
ADRA2C	4p16.3	104250	Adrenoceptor alpha 2C	Congestive heart failure and beta-blocker response
MSX1	4p16.2	142983	msh homeobox 1	Tooth agenesis, selective, 1, with or without orofacial cleft, ectodermal dysplasia 3, Witkop type, orofacial cleft 5
EVC2	4p16.2	607261	EvC ciliary complex subunit 2	Diseases: Weyers acrofacial dysostosis, Ellis-van Creveld syndrome
EVC	4p16.2	604831	EvC ciliary complex subunit 1	Diseases: Weyers acrofacial dysostosis, Ellis–van Creveld syndrome
WFS1	4p16.1	606201	Wolframin ER transmembrane glycoprotein	Cataract 41, diabetes mellitus, noninsulin-dependent (NIDDM), Wolfram syndrome 1, deafness, autosomal dominant 6/14/38, Wolfram-like syndrome, autosomal dominant
HMX1	4p16.1	142992	H6 family homeobox 1	Oculoauricular syndrome
SLC2A9	4p16.1	606142	Solute carrier family 2 member 9	Hypouricemia, renal, 2 (RHUC2)
DRD5	4p16.1	126453	Dopamine receptor D5	Attention deficit-hyperactivity disorder (ADHD), blepharospasm, primary benign
RAB28	4p15.33	612994	RAB28, member RAS oncogene family	Cone-rod dystrophy 18
NKX3-2	4p15.33	602183	NK3 homeobox 2	Spondylomegaepiphyseal-metaphyseal dysplasia
CC2D2A	4p15.32	612013	Coiled-coil and C2 domain containing 2A	COACH syndrome, Meckel syndrome 6, Joubert syndrome 9
PROM1	4p15.32	604365	Prominin 1	Stargardt disease 4, macular dystrophy, retinal, 2, retinitis pigmentosa 41, cone-rod dystrophy 12
TAPT1	4p15.32	612758	Transmembrane anterior posterior transformation 1	Osteochondrodysplasia, complex lethal, Symoens-Barnes-Gistelinck type
QDPR	4p15.32	612676	Quinoid dihydropteridine reductase	Diseases: hyperphenylalaninemia, BH4-deficient, C
STS	Xp22.31	300747	Steroid sulfatase	Ichthyosis, X-linked

## Data Availability

The data used to support the findings of this study are included within the article.
